# Protective Role of the *PG1036-PG1037-PG1038* Operon in Oxidative Stress in *Porphyromonas gingivalis* W83

**DOI:** 10.1371/journal.pone.0069645

**Published:** 2013-08-19

**Authors:** Leroy G. Henry, Wilson Aruni, Lawrence Sandberg, Hansel M. Fletcher

**Affiliations:** 1 Division of Microbiology and Molecular Genetics, School of Medicine, Loma Linda University, Loma Linda, California, United States of America; 2 Division of Biochemistry, School of Medicine, Loma Linda University, Loma Linda, California, United States of America; University of Florida, College of Dentistry & The Emerging Pathogens Institute, United States of America

## Abstract

As an anaerobe, *Porphyromonas gingivalis* is significantly affected by the harsh inflammatory environment of the periodontal pocket during initial colonization and active periodontal disease. We reported previously that the repair of oxidative stress-induced DNA damage involving 8-oxo-7,8-dihydroguanine (8-oxoG) may occur by an undescribed mechanism in *P. gingivalis*. DNA affinity fractionation identified PG1037, a conserved hypothetical protein, among other proteins, that were bound to the 8-oxoG lesion. *PG1037* is part of the *uvrA-PG1037-pcrA* operon in *P. gingivalis* which is known to be upregulated under H_2_O_2_ induced stress. A PCR-based linear transformation method was used to inactivate the *uvrA* and *pcrA* genes by allelic exchange mutagenesis. Several attempts to inactivate *PG1037* were unsuccessful. Similar to the wild-type when plated on Brucella blood agar, the *uvrA* and *pcrA*-defective mutants were black-pigmented and beta-hemolytic. These isogenic mutants also had reduced gingipain activities and were more sensitive to H_2_O_2_ and UV irradiation compared to the parent strain. Additionally, glycosylase assays revealed that 8-oxoG repair activities were similar in both wild-type and mutant *P. gingivalis* strains. Several proteins, some of which are known to have oxidoreducatse activity, were shown to interact with PG1037. The purified recombinant PG1037 protein could protect DNA from H_2_O_2_-induced damage. Collectively, these findings suggest that the *uvrA-PG1037-pcrA* operon may play an important role in hydrogen peroxide stress-induced resistance in *P. gingivalis*.

## Introduction

*Porphyromonas gingivalis*, a black-pigmented Gram-negative anaerobic bacterium has been recognized as a major pathogen in adult periodontitis and is associated with other systemic diseases including cardiovascular disease and rheumatoid arthritis [1], [2]. As a secondary colonizer and a “keystone” pathogen, *P. gingivalis* even when present in low numbers, is able to manipulate the host immune system, thus eliciting a major effect on the composition of the oral microbial community which significantly contributes and may ultimately be responsible for the pathology of periodontitis [3].

Colonization, growth and survival of *P. gingivalis* in the inflammatory microenvironment of the periodontal pocket are important attributes that are vital for its pathogenesis. This is facilitated by many virulence factors, which include adhesion proteins such as hemagglutinins, that can mediate its interaction with host tissues and other commensal bacteria [4]. *P. gingivalis* also expresses proteases known as gingipains, considered to be major virulence factors, that are involved in several processes known to be important for bacterial growth and can compromise cellular integrity and host cell functions by several mechanisms triggered by, for example, inactivation of cytokines, platelet aggregation, and apoptosis [5]. Furthermore, the role of these gingipains in heme accumulation on the cell surface is important in oxidative stress resisitance [5]. Although there is evidence that *Fusobacterium* is important in creating a reduced environment for *P. gingivalis* [6], [7], several genes including superoxide dismutase (*sod*), rubrerythrin (*rbr*), and DNA binding proteins (*dps*) have been shown to play a role in oxidative stress resistance in this organism [8]–[10]. Oxidative stress conditions can generate and cause the accumulation of O_2_^*^, H_2_O_2_ and hydroxyl radicals [11]–[13]. Of these species, H_2_O_2_ is extremely harmful as it easily penetrates membranes and diffuses through cells [14]. It has the ability to form adducts (hydrogen-bonded chelate structures) with various cell constituents such as amino acids (e.g. histidine, alanine, glycine, aspartic acid), succinic acid and DNA bases, which act as H_2_O_2_ carriers [15]. These characteristics allow H_2_O_2_ to act at sites distinct from the site of its production, enhancing its damaging potential. The metabolism of anaerobes usually depends on metabolic schemes built around enzymes that react easily with oxygen [16]. Hydrogen peroxide formation could therefore be one of the main sources of toxicity for anaerobic microorganisms.

Major targets of these oxidants include DNA, cellular membranes, metalloproteases and transcription factors [16]–[20]. In bacteria, the damage to DNA appears to be the most significant [reviewed in [13]]. Host inflammatory responses and by-products of the normal metabolism of all cells can produce oxygen radicals. Among the main lesions produced in DNA by ROS is an oxidized form of guanine, 8-oxo-7,8-dihydroguanine (8-oxo-G), which has a strong mutagenic potential. This modified base, when present on the template strand, induces the incorporation of an adenine opposite it during DNA replication, leading to G:C-to-T:A transversions [21], [22] that can be deleterious to the cell. Removal of 8-oxoG, first characterized for *Escherichia coli*, appears to occur mostly by the base excision repair (BER) process involving the foramidopyrimidine glycosylase (Fpg) enzyme encoded by the *mutM* gene [23], [24]. Alternatively, nucleotide excision repair (NER) is different from all the other forms of DNA repair in its ability to act on a wide variety of substrates [25], [26]. It is mediated by the products of *uvrABC*, *uvrD*, *polA* and *lig* genes and recognizes distortions in DNA caused by bulky adducts that also alter the chemistry of the DNA.

Repair of the 8-oxoG lesion in *P. gingivalis* was previously reported to occur by a non-BER mechanism [23]. This was consistent with the absence of any FPG homologue in *P. gingivalis* [27] (http://www.ncbi.nlm.nih.gov/). To further evaluate if NER played a role in this repair activity, the *uvrB* gene, the central component of bacterial NER, was inactivated [28]. In contrast to the wild-type *P. gingivalis* W83, the *uvrB*-deficient mutant FLL144 was significantly more sensitive to UV irradiation. The sensitivity of *P. gingivalis* FLL144 (*uvrB::ermF-ermAM*) to H_2_O_2_-induced oxidative stress was similar to the parent strain [28]. Moreover, the enzymatic removal of 8-oxoG was unaffected by the inactivation of the *uvrB* gene. These results suggested that the *uvrB* gene in *P. gingivalis* may not be involved in the removal of 8-oxoG and that another yet unidentified mechanism may be employed in its repair. In this study, we report that the *uvrA-PG1037-pcrA* operon may play an important role in hydrogen peroxide stress-induced resistance in *P. gingivalis*. PG1037, a putative zinc finger protein that can bind to the 8-oxoG lesion can interact with other proteins that have oxidoreductase properties. In addition, PG1037 contains two putative peroxidase domains, which raises questions on its ability to scavenge ROS and protect DNA for oxidative stress-induced damage.

## Materials and Methods

### Bacterial strains and culture conditions

Strains and plasmids used in this experiment are listed in Table 1. *P. gingivalis* strains were grown in brain heart infusion (BHI) broth (Difco Laboratories, Detroit, MI) supplemented with hemin (5 µg/ml), vitamin K (0.5 µg/ml) and cysteine (0.1%). *E. coli* strains were grown in Luria-Bertani broth (LB) [Sambrook et al., 1989 [29]]. L-cysteine was omitted from broth for experiments in which cells were treated with hydrogen peroxide. For BHI plates, broth was supplemented with agar (20 g/L). For BHI blood agar plates, broth was supplemented with defibrinated sheep blood (5%) and agar (1%). Unless otherwise stated, all cultures were incubated at 37°C. *P. gingivalis* strains were maintained in an anaerobic chamber (Coy Manufacturing, Ann Arbor, MI) in 10% H_2_, 10% CO_2_, 80% N_2_. Growth rates for *P. gingivalis* and *E. coli* strains were determined spectrophotometrically (optical density at 600 nm). For selection, the antibiotics erythromycin and carbenicillin was added at 10 µg/ml and 50 µg/ml respectively.

**Table 1:** Plasmids and bacterial strains used in this study.

Strains and Plasmids	Phenotype/Description	*Source*
***Plasmids***		
pVA2198	Sp^r^, ermF-ermAM	[*31*]
pFLL143	*PG1037* in pET102/D-TOPO®	
***Bacterial strains***		
*Porphyromonas gingivalis*		
W83	Wild type	[*32*]
FLL92	*vimA* defective (*vimA::ermF-ermAM*)	[*78*]
FLL144	*uvrB* defective (*uvrB::ermF-ermAM*)	[*28*]
FLL145	*uvrA* defective (*ΔuvrA*)	*This study*
FLL146	*pcrA* defective (*ΔpcrA*)	*This study*
FLL147	*uvrA* with *ermF*- terminator (*ΔuvrA*-T)	*This study*
FLL148	*uvrA* with *ermF* in the reverse orientation (*ΔuvrA*-R)	*This study*
FLL149	*uvrA* complemented	*This study*
FLL150	*pcrA* complemented	*This study*
*E.coli*		
Top10	F-*mcr*A D (*mrr*-*hsd*RMS-*mcr*BC) f80*lac*ZD M15 D*lac*X74 *rec*A1 ara139 D (*ara-leu*)7697 *gal*U *gal*K *rps*L (StrR) *end*A1 *nup*G	*Invitrogen*
*BL21Star™(DE3)*	*F^−^ompT hsdS_B_ (r_B_^−^m_B_^−^) gal dcm rne131(DE3)*	*Invitrogen*

### PCR-based linear transformation to construct *P. gingivalis* FLL145 (Δ*uvrA*), FLL146 (Δ*pcrA*), FLL147 (*ΔuvrA-*T), FLL148 (*ΔuvrA*-R) mutants

Long PCR-based fusion of several fragments was performed as described previously [30]. The primers used in this study are listed in Table 2. Briefly, 1 kb flanking fragments both upstream and downstream of the target genes were PCR amplified from *P. gingivalis* W83 chromosomal DNA in individual 50 µl reaction mixtures containing 1 µl of template DNA (0.5 µg), a 1 µM concentration of each primer, 25 µl high-fidelity PCR master enzyme mix (Roche, Indianapolis, IN), and distilled water. The *ermF* cassette was amplified from the pVA2198 [31] plasmid with oligonucleotide primers that contained overlapping nucleotides for the upstream and downstream fragments. These three fragments were fused together using the forward primer of the upstream fragment and the reverse primer of the downstream fragment. The fusion PCR program consisted of 1 cycle of 5 min at 94°C, followed by 30 cycles of 30 seconds at 94°C, 30 seconds at 55°C, and 4 min at 68°C, with a final extension of 5 min at 68°C. This PCR-fused fragment was used to transform *P. gingivalis* W83 by electroporation as described previously [32]. The cells were plated on a BHI agar containing 10 µg/ml of erythromycin and incubated at 37°C for 7 days. The correct gene replacement in the erythromycin-resistant mutants was confirmed by colony PCR and DNA sequencing.

**Table 2:** Primers used in this study.

Primers	Sequence (5′-3′
**Primers for FLL145 construction**	
**FLL145_F1**	CTATTCCCTATTTTCAGCCGAA
**FLL145_F2**	TTCGTAGTACCTGGAGGGAATAATCGAATTCTGCAGACAATATATAATGTAAAG
**FLL145_R1**	GTCATTTATTCCTCCTAGTTAGTCATCTTTTACGTTTATAACTGTATCGTGCAT
**FLL145_R2**	CGTACAAGGGGGAACATTATTAG
**Primers for FLL146 construction**	
**FLL146_F1**	TGCGTGTTGGACGAGAAG
**FLL146_F2**	TTCGTAGTACCTGGAGGGAATAATCTATCCAAAGTGGAAAAAGATTCGATCTGAA
**FLL146_R1**	GTCATTTATTCCTCCTAGTTAGTCATTCAATGATGAGAGATAATCTTCGGACAT
**FLL146_R2**	GAGAAAGCCGTGCAGGAG
**Primers for FLL147 construction**	
**FLL147_F1**	CTATTCCCTATTTTCAGCCGAA
**FLL147_F2**	AAATTTGTAATTAAGAAGGAGTGATTACGAATTCTGCAGACAATATATAATGTAAAG
**FLL147_R1**	GTCATTTATTCCTCCTAGTTAGTCATCTTTTACGTTTATAACTGTATCGTGCAT
**FLL147_R2**	CGTACAAGGGGGAACATTATTAG
**Primers for FLL148 construction**	
**FLL148_F1**	CTATTCCCTATTTTCAGCCGAA
**FLL148_F2**	TACCTTATTCCTCCTAGTTAGTCAGAATTCTGCAGACAATATATAATGTAAAG
**FLL148_R1**	TTCGTAGTACCTGGAGGGAATAATCTCTTTTACGTTTATAACTGTATCGTGCAT
**FLL148_R2**	CGTACAAGGGGGAACATTATTAG
**Primers for ermF without a terminator, in the forward orientation**	
**ErmF_F1**	TGACTAACTAGGAGGAATAAATGACAAAAAAGAAATTGCCCG
**ErmF_R1**	GATTATTCCCTCCAGGTACTACGAAGGATGAAATTTTTCA
**Primers for ermF with a terminator, in the forward orientation**	
**ErmF_Term_F1**	TGACTAACTAGGAGGAATAAATGACAAAAAAGAAATTGCCCG
**ErmF_Term_R1**	GTAATCACTCCTTCTTAATTACAAATTT
**PT-PCR primers**	
**PG1036_F**	ATGCACGATACAGTTATAAACGTAAAA
**PG1036_R**	TATATTGTCTGCAGAATTCATGTGTAC
**PG1037_F**	ATGAAAGAACAGAAATTGTCTAATCGG
**PG1037_R**	CGATGCCCCGACAATCATA
**PG1038_F**	ATGTCCGAAGATTATCTCTCATCAT
**PG1038_R**	GATCGAATCTTTTTCCACTTTGG
**VimA_F**	
**VimA_R**	
**16S_rRNA_F**	AGGCAGCTTGCCATACTGCG
**16S_rRNA_R**	ACTGTTAGCAACTACCGATG
**PT-PCR internal primers**	
**PG1036_INT_F**	GCAAACGTATAGCGGAGAGC
**PG1036_INT_R**	CCACTTCACCACCATGTCTG
**PG1037_INT_F**	ACCGATACCGAGTTGCATTC
**PG1037_INT_R**	CACATCATCAGGCAGATTGG
**PG1038_INT_F**	TCGGAGTACACAGAGCATCG
**PG1038_INT_R**	ATGGGAAGTTCGTCACCTTG
**Primers for ORF of ** ***PG1037***	
**PG1037_ORF_F**	CACCCATGAAAGAACAGAAATTGTCTAAT
**PG1037_ORF_R**	CGATGCCCCGACAATCATA
**Primers for ** ***uvrA*** ** complementation**	
**FLL149_F**	AAC GGG ATC GCG CCA CTC CTT CAA ACG AGC
**FLL149_R**	GTG GAT GCA GCC ATC CGG CCT ATT CCC TAT TTT CA
**Primers for ** ***pcrA*** ** complementation**	
**FLL150_F**	TGC GTG TTG GAC GAG AAG AGA CGG CAC GCT TT
**FLL150_R**	GAG AAA GCC GTG CAG GAA AAT GGT ATG ACG ATC C

### Reverse transcriptase Polymerase Chain Reaction (RT-PCR) analysis of DNase treated RNA extracted from *P. gingivalis*

Total RNA was extracted from *P. gingivalis* W83 and isogenic mutant strains grown to mid-log phase (OD_600_ of 0.7) using the RiboPure™ kit (Ambion, Austin, TX). Reverse transcription and PCR amplification was performed with a Perkin-Elmer Cetus DNA thermal Cycler (Perkin Elmer Corporation, Norwalk, CT). The primers used in this study are listed in Table 2. The final products were analyzed by electrophoresis in 1% agarose.

### Gingipain activity assay

*P. gingivalis* extracellular protein extracts were prepared as previously reported [33]. The presence of Arg-X and Lys-X activity was determined using a microplate reader (Bio-Rad Laboratories, Hercules, CA) according to the methods of Potempa et al., [34].

### Sensitivity to hydrogen peroxide and UV irradiation

*P. gingivalis* strains were grown to early log phase [OD_600_ 0.2] in BHI broth. Hydrogen peroxide at concentrations of 0.25 mM was then added to the cell cultures and further incubated for 30 h. The optical density at 600 nm was then measured at 4 h intervals over a 24 h period. Cell cultures without hydrogen peroxide were used as controls. UV sensitivity test was done as previously reported [32].

### Complementation of the *P. gingivalis* FLL145 (Δ*uvrA*), FLL146 (Δ*pcrA*), FLL147 (*ΔuvrA-*T), FLL148 (*ΔuvrA*-R) mutants

PCR mediated gene replacement was used to complement the *uvrA* or *pcrA* defect. The ORF of *uvrA* [>gi|34539880:1098576-1101479 *P. gingivalis* W83 chromosome (http://www.ncbi.nlm.nih.gov/)] or *pcrA* [>gi|34539880:1102884-1105181 *P. gingivalis* W83 chromosome (http://www.ncbi.nlm.nih.gov/)] with 1 kb flanking regions both upstream and downstream was amplified from W83 chromosomal DNA using oligonucleotide primers (Table 2). Electroporation of the amplified fragments into *P. gingivalis uvrA* or *pcrA*-defective mutants were performed as previously reported [35]. Electroporated cells were incubated for 12 h in 1 ml of BHI broth then treated with 0.25 mM hydrogen peroxide for 10 min and plated on BHI agar without antibiotics. The plates were incubated for 7 days at 37°C. Colony PCR and DNA sequencing were used to confirm the appropriate gene replacement in *P. gingivalis uvrA* and *pcrA*-defective mutants. RT-PCR was also performed on complemented strains to confirm gene expression of the *PG1036* (*uvrA*) or *PG1038* (*pcrA*) genes.

### Preparation of crude bacterial extracts

Bacterial protein extracts were prepared as previously described [23]. Briefly, *P. gingivalis* cultures were grown overnight in BHI. A 1/10 dilution of each bacterial strain was made in fresh pre-warmed BHI medium and grown to an OD_600_ of 0.6. *E. coli* was grown in a similar manner under aerobic conditions. The cell pellets were collected by centrifugation (9,000× *g* for 10 min at 4°C) treated with protease inhibitors, resuspended in 5 ml of 50 mM Tris-HCl (pH 8.0) lysis buffer, and subjected to French Pressure Cell Press (American Instrument Company, Silver Spring, MD). Cell debris was removed by centrifugation at 12,000× *g* for 20 min at 4°C and then by ultra-centrifugation at 45,000× *g* for 1 h at 4°C. The protein concentration of the supernatant was determined using the BCA protein assay kit (Pierce, Rockford, IL).

### Cloning of *PG1037* into an expression vector

Oligonucleotide primers specific for the ORF of the *PG1037* gene were synthesized (see Table 2) and used in PCR amplification of the *PG1037* gene as previously described [32]. The fragment carrying the *PG1037* gene was cloned into pET102/D-TOPO® expression plasmid (Invitrogen, Carlsbad, CA) carrying coding for an N-terminal His-Patch (HP)-thioredoxin fusion tag. The recombinant plasmid, designated pFLL143, was transformed into competent BL21(DE3)pLysS *Escherichia coli*. The orientation was determined by restriction endonuclease digestion. The nucleotide sequence of the insert in pFLL143 was analyzed by DNA sequencing to rule out any mutations.

### Expression and purification of rPG1037

*E. coli* BL21(DE3)pLysS carrying pFLL143 was grown to exponential phase (OD_600_∼0.5) in Luria–Bertani broth in the presence of carbenicillin (50 µg/ml). IPTG to a final concentration of 0.75 mM was added and the culture was further incubated at 37°C with shaking for a 4 h. Cells were harvested by centrifugation and subjected to French Pressure Cell Press (American Instrument Company, Silver Spring, MD). Cell debris was removed by centrifugation at 12,000× *g* for 20 min at 4°C and then by ultra-centrifugation at 45,000× *g* for 1 h at 4°C. The supernatant was further purified using HisPur Cobalt Spin Columns (Pierce, Rockford, IL) and by manufacturer's standards determined to be >90% pure. The presence of the poly-histidine tag was confirmed using the GelCode 6×His Protein Tag kit according to the manufacturer's instructions (Pierce, Rockford, IL).

### Bioinformatics analysis

The amino acid sequences were retrieved from the Oralgen database [Los Alamos National Laboratory; http://www.oralgen.lanl.gov] and aligned using Bioedit (http://www.mbio.ncsu.edu/bioedit/bioedit.html). The amino acid sequences were analyzed using ClustralW version 2.0. The secondary structure prediction and modeling of the protein was performed using the Modeller 9v8 program [36]. Threading was performed using the HHpred interactive server for protein homology detection and structure prediction [37]. The models were then validated using WHATIF program [38].

### Oligonucleotide labeling and annealing procedures

Oligonucleotide fragments (see Table 3) used in this study was synthesized by Synthegen (Houston, TX). Labeling and annealing procedures were performed as previously described [23].

**Table 3:** Oligonucleotides used in this study.

**O1**
5′-GACTACGTACTGTTACGGCTCCATC**X**CTACCGCATTCAGGCCAGATCTGC-3′
3′-CTGATGCATGACAATGCCGAGGTAG**C**GATGGCGTAAGTCCGGTCTAGACG-5′
**O2**
5′-GACTACGTACTGTTACGGCTCCATC**G**CTACCGCATTCAGGCCAGATCTGC-3′
3′-CTGATGCATGACAATGCCGAGGTAG**C**GATGGCGTAAGTCCGGTCTAGACG-5′
**X:** 8-oxoG (O1)

### Glycosylase assay

Labeled and annealed oligonucleotides (10 pmol) were incubated at 37°C for 20 min with *P. gingivalis* or *E. coli* cell extracts (2 µg) while in other experiments, labeled and annealed oligonucleotides were incubated with rPG1037 protein for 20 min and 2 mM H_2_O_2_ for 10 min in a 1× enzyme buffer supplied with Formamidopyrimidine-DNA glycosylase (Fpg) enzyme (Trevigen Inc., Gaithersburg, MD). An equal volume of loading buffer (98% formamide, 0.01 M EDTA, 1 mg/ml xylene cyanol, and 1 mg/ml bromophenol blue) was added to stop the reaction. 20 pmol of competitor oligonucleotide was added to each reaction mix and heated to 95°C for 5 min to denature the duplex, after which it was resolved by gel electrophoresis. As controls, Fpg and Ung control reactions were performed according to Liu et al., [39]. Briefly, 10 pmol of the specific oligonucleotide was incubated with 1 unit of the enzyme at 37°C for 1 h in reaction buffers provided by the manufacturers.

### Gel electrophoresis and analysis of cleavage

Reaction samples were loaded onto a 20% denaturing polyacrylamide gel (7 M urea) and run for 2 h at 500 V in 1× Tris-Borate-EDTA (TBE) buffer. The resulting bands corresponding to the cleavage products and uncleaved substrate was visualized using a Molecular Dynamics Phosphor Imager (Amersham, Biosciences, Piscataway, NJ) and ImageQuant 5.0 software.

### DNA protection from Fenton Chemistry-mediated DNA damage

The ability of rPG1037 to protect DNA from oxidative damage in vitro was assessed as previously described [40]. Purified pUC19 plasmid DNA was incubated with varying concentrations of rPG1037 in the presence of FeSO4 (50 uM) in Tris buffer (20 mM, pH 7.8) and double distilled water for a final volume of 18 µl. The reaction was allowed to incubate at room temperature for 5 min. 2 µl hydrogen peroxide (880 mM) was added to the appropriate reactions and allowed to further incubate for 30 min. 2 µl of 10× DNA loading dye was added to the mixture and the entire reaction loaded on a 1% agarose gel. The gel was stained with ethidium bromide after electrophoresis and visualized on a UVP photo documentation system (Upland, CA).

### Protein–Protein interaction studies

Approximately 2000 µg of the purified rPG1037 protein was incubated with the Ni-NTA-linked magnetic beads. The beads with attached rPG1037 were washed with wash/interaction buffer (50 mM NaH_2_PO_4_, 300 mM NaCl, 20 mM imidazole and 0.005% Tween 20 pH 8.0) and incubated with cell lysates or extracellular fractions from *P. gingivalis* W83 or FLL92 (*vimA::ermF-ermAM*). As a negative control, the lysates or extracellular fractions from *P. gingivalis* were incubated with the magnetic beads without the attached rPG1037. After incubation, the unbound proteins were eliminated by three washings with wash/interaction buffer. Proteins were eluted off the beads using 50 µl of elution buffer (50 mM NaH_2_PO_4_, 300 mM NaCl, 250 mM imidazole and 0.005% Tween 20, pH 8.0).

### Identification of protein that can physically interact with rPG1037

SDS-PAGE was performed with a 1× SDS-Page running buffer (BioRad, Hercules, CA) according to manufacturer's instructions. The gels were run for 1.5 cm then stained with SimplyBlue™ Safe Stain (Invitrogen, Carlsbad, CA) to visualize bands. After briefly destaining in water, the gel was cut into four equally-spaced slices (∼2 mm each) for Trypsin digestion. As a control, a gel slice was cut from a blank region of the gel and processed in parallel with the sample. The excised gel pieces were dehydrated in acetonitrile and dried in a vacuum centrifuge for 30 min. The proteins were reduced in 20 ul of 20 mM dithiothreitol (DTT) in 100 mM NH_4_HCO_3_ (sufficient to cover the gel pieces) for 1 h at 60°C. After cooling to room temperature, the DTT solution was replaced with an alkylating solution consisting of 20 µl of 200 mM iodoacetamide in 100 mM NH_4_HCO_3_. After 30 min incubation at ambient temperature in the dark, the gel pieces were washed twice with 150 µl 100 mM NH_4_HCO_3_, finely minced with a flame sealed polypropylene pipette tip, dehydrated by the addition of acetonitrile and then dried in a vacuum centrifuge. The gel pieces were rehydrated and incubated overnight at 37°C in 20 µl digestion buffer containing 0.1 µg of trypsin MS grade (Promega, Madison, WI) in 50 mM acetic acid with equal parts of 100 mM NH_4_HCO_3._ The digestion was stopped with 10 µl, 5% formic acid. The digest solution (aqueous extraction) was transferred into a clean 0.65 ml siliconized tube. To the gel pieces, 30 µl of 50% acetonitrile with 5% formic acid was added, vortexed for 30 min, centrifuged, and then sonicated for 5 min. This process was repeated and both aqueous extractions were combined and concentrated to 10 µl in a vacuum centrifuge. Peptides were extracted and purified using standard C_18_ ZipTip technology following the manufacturer's directions (Millipore, Bedford, MA). The final volume of each preparation was 20 µl in 0.1% formic acid.

### Mass Spectrometry and data analysis

The extracted peptides from each gel piece were analyzed using an LCQ Deca XP plus system (Thermo Finnigan, San Jose, CA) using nano-electrospray technology (New Objectives, Woburn, MA). MS and MS/MS analyses were accomplished with a 4-part protocol that consisted of one full MS analysis (from 150 to 2000 m/z) followed by 3 MS/MS events using data dependent acquisition, where the first most intense ion from a given full MS scan was subjected to CID followed by the second and third most intense ions [41]. With the cycle repeating itself, the nanoflow buffer gradient was extended using a 0 to 60% acetonitrile gradient from buffer B (95% acetonitrile with 0.1% formic acid) developed against buffer A (2% acetonitrile with 0.1% formic acid) at a flow rate of 250–300 nl/min with a final 5 min 80% bump of buffer B before re-equilibration. A Scivex 10 port automated valve (Upchurch Scientific, Oak Harbor, WA) together with a Michrom nanotrap column (Michrom Bioresources, Auburn, CA) were used to move the 20 ul sample from the autosampler to the nanospray unit. Data was collected with the Xcalibur software (Thermo Electron) and screened with Bioworks 3.1. Peptide tandem mass spectra were processed by Turbo SEQUEST software (v.27 (rev. 14, (c) 1999–2002)) [42], [43] and analyzed using the *P. gingivalis* fasta database available from NCBI (Jan 2008). Proteome Software's SCAFFOLD 1.7. meta analysis software together with X! TANDEM (thegpm.org) was then used to statistically validate the peptide and protein findings of SEQUEST [44]. Protein identity was confirmed when at least two different peptides were present at least at 95% probability and the protein probability was also at 95% or better. Individual peptide matches were then confirmed with the BLAST database at http://www.oralgen.lanl.gov.

## Results

### PG1037 is part of the uvrA-PG1037-pcrA operon in P. gingivalis

**Figure 1:**
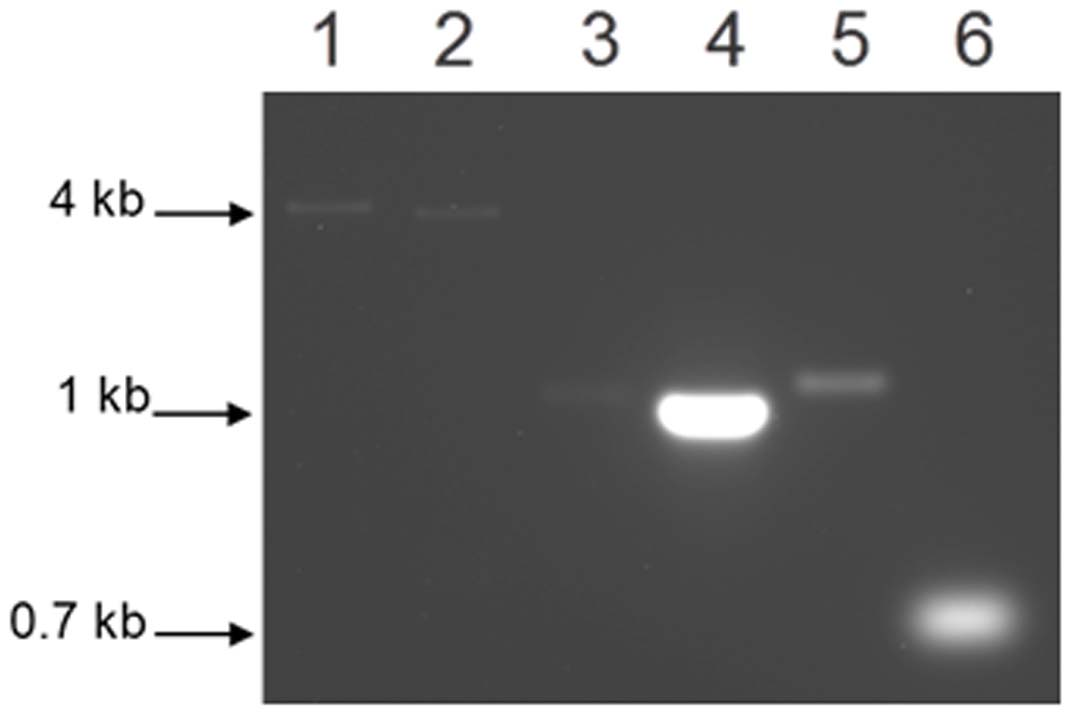
RT-PCR analysis of *P. gingivalis* W83. DNase treated total RNA extracted from *P. gingivalis* W83 and subjected to RT-PCR. Lane 1, amplification of 4.3 kb *uvrA-PG1037* fragment; Lane 2, amplification of 3.7 kb *PG1037-pcrA* fragment; Lanes 3, 4 and 5, intragenic specific primers for *uvrA*, *PG1037* and *pcrA* genes amplified a 1.2 kb, 1.0 kb and 1.3 kb fragments respectively; Lane 6, amplification of 0.7 kb 16S fragment.

Neither BER nor NER, as observed in other bacteria, appear to be involved in the repair of the 8-oxoG lesion in *P. gingivalis* [23], [28]. DNA affinity fractionation however identified PG1037 (a hypothetical protein of unknown function) that was observed to bind only to the 8-oxoG oligonucleotide fragment [28]. Furthermore, transcriptional profiling of *P. gingivalis* showed that the *PG1036 (uvrA)* and *PG1038 (pcrA)* genes were upregulated in cells exposed to hydrogen peroxide-induced stress [45]. *In silico* analysis of *PG1036 (uvrA)*, *PG1037* and *PG1038 (pcrA)* suggests they may be part of the same transcriptional unit. To confirm that the gene encoding PG1037 was part of a three gene operon, total RNA was isolated from the wild-type *P. gingivalis* W83 grown to mid-log phase [OD_600_ of 0.7]. If these genes are transcribed as a single transcriptional unit, a 4.3 kb fragment should be amplified using a 5′ oligonucleotide primer from *PG1036* and a 3′ primer for *PG1037* (Table 2). As shown in Figure 1, the expected 4.3 kb fragment was observed only when reverse transcriptase was used in the RT-PCR reactions (Lane 1). A 3.7 kb *PG1037-pcrA* fragment was also amplified using oligonucleotide primers PG1037_F and PG1038_R (Table 2) [Figure 1, Lane 2]. Additionally, as shown in Figure 1, specific intragenic primers for *uvrA*, *PG1037* and *pcrA* amplified 1.2 kb, 1.0 kb and 1.3 kb fragments respectively. No amplification was observed in the wild-type *P. gingivalis* strain in the absence of reverse transcriptase (data not shown). As a control, a 0.7 kb fragment was also amplified using 16S-specific primers. Taken together, these data confirm that the gene encoding PG1037 is part of the *uvrA-PG1037-pcrA* operon.

### Inactivation of genes in the *uvrA-PG1037-pcrA* operon

**Figure 2:**
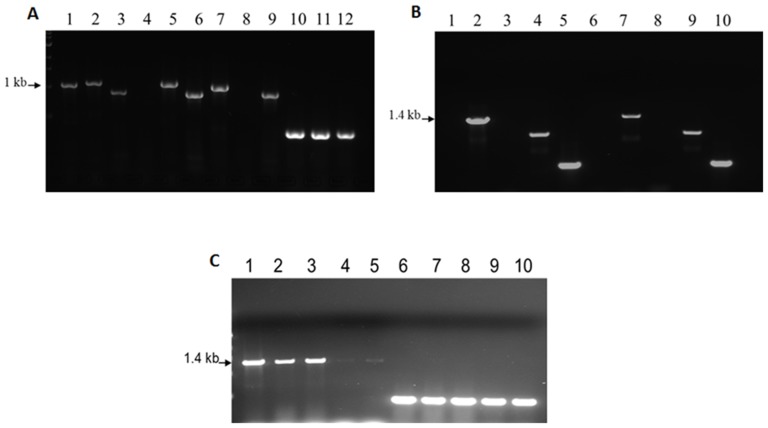
RT-PCR analysis of *P. gingivalis* W83. DNase treated total RNA was extracted from *P. gingivalis* W83 and isogenic mutants (FLL145, FLL146, FLL147 and FLL148) then subjected to RT-PCR. In **Panel A**, lanes 1, 2 and 3 shows expression of a 1.2 kb, 1.3 kb and 0.9 kb uvrA, pcrA and vimA fragments respectively in wild-type *P. gingivalis* W83; lane 4 shows no expression of the 1.2 kb uvrA fragment in the FLL145 mutant but shows expression of pcrA and vimA fragments as seen in lanes 5 and 6; in the FLL146 mutant, lanes 7 and 9 shows expression of uvrA and vimA fragments respectively but no expression of pcrA fragments is seen in lane 8; as a positive control, lanes 10, 11 and 12 shows expression of 16S ribosomal RNA. In **Panel B**, neither lanes 1 or 3 and 6 or 8 showed amplification of uvrA and pcrA fragments in FLL147 and FLL148 mutants respectively; lanes 2 and 7 showed amplification of *PG1037* in FLL147 and FLL148 mutants respectively; lanes 4 and 9 shows amplification of vimA in FLL147 and FLL148 mutants respectively; lanes 5 and 10 shows amplification of 16S ribosomal RNA. In **Panel C**, lanes 1–5 shows that *PG1037* is expressed in *P. gingivalis* W83 and all isogenic mutants; lanes 6–10 shows amplification of vimA in *P. gingivalis* W83 and all isogenic mutants.

Isogenic mutants of *P. gingivalis* defective in PG1036 and PG1038 were constructed by allelic exchange mutagenesis. Because there is high transformation efficiency using linear PCR generated fragments [35], PCR was used to fuse the upstream and downstream fragments of the target gene to the *ermF* cassette without a transcription terminator. This generated a 3 kb-length fragment which was then electroporated into *P. gingivalis* W83. Isogenic mutants defective in the *uvrA* and *pcrA* genes were confirmed by colony PCR and sequencing of chromosomal DNA extracted from the isogenic mutants (data not shown). Several unsuccessful attempts were made to create a defect in the *PG1037* gene. To rule out polar mutations arising from the inactivation of *uvrA* and *pcrA* genes, RT-PCR was used to amplify genes in the *uvrA-PG1037-pcrA* operon. While there was no expression of the *uvrA* or *pcrA* genes in the FLL145 (Δ*uvrA*) or FLL146 (Δ*pcrA*) isogenic mutants respectively, the downstream genes were still transcribed (Figure 2.A).

To further investigate the role of *PG1037*, isogenic mutants defective in *uvrA* were created that would have a polar effect on the downstream genes. This was done by inserting the *ermF* cassette with its terminator or the *ermF* cassette in the reverse orientation to create the FLL147 (Δ*uvrA*-T) and FLL148 (Δ*uvrA*-R) mutants respectively (Table 2). In the FLL147 (Δ*uvrA*-T) and FLL148 (Δ*uvrA*-R) isogenic mutants, using RT-PCR, there was no expression of the downstream *pcrA* gene (Figure 2.B) however expression of the 1.4 kb *PG1037* fragment was observed in both strains (Figure 2.C). Taken together it is likely that *PG1037* is an essential gene with its own promoter which allows for independent expression.

### Gingipain activity is affected by inactivation of genes in the *uvrA-PG1037-pcrA* operon

**Figure 3:**
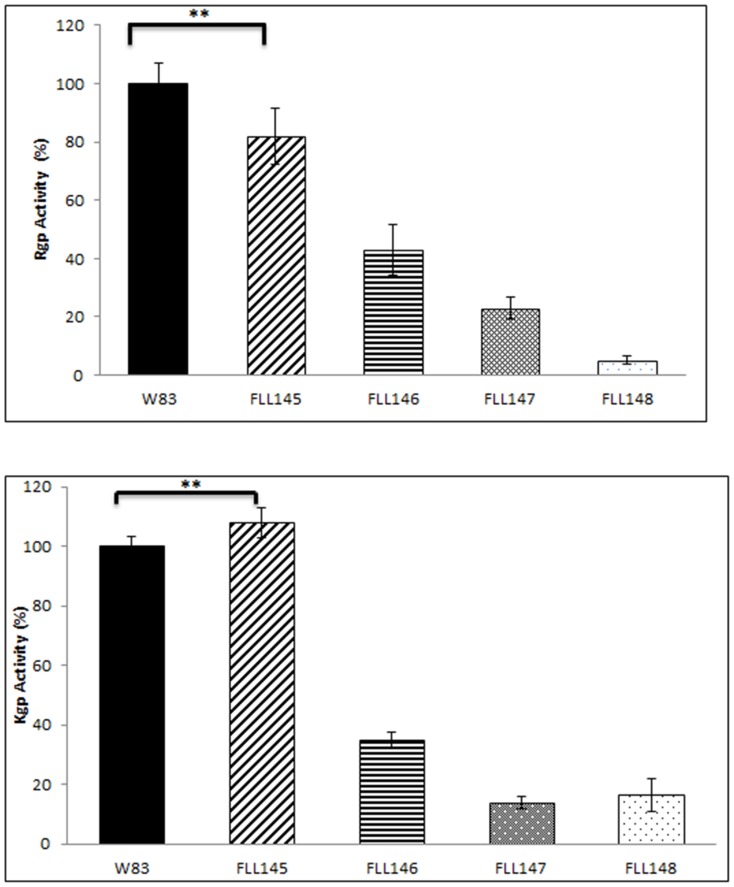
Proteolytic activity of *P. gingivalis* mutants. *P. gingivalis* strains were grown to late log phase OD_600_ of 1.2 in 50 ml of BHI broth supplemented with hemin and vitamin K. **Panel A**; whole cell culture was analyzed for Rgp (BAPNA) activity (**P≤0.01). **Panel B**; whole cell culture was analyzed for Kgp (ALNA) activity. The results shown are representative of 3 independent experiments performed in triplicate (*P≤0.01).

Gingipains, which are both extracellular and cell membrane associated, are major virulence factors of *P. gingivalis* [5], [46]. Gingipains can also play a role in oxidative stress resistance [5], [47]. In order to identify whether the *uvrA-PG1037-pcrA* operon was involved in gingipain regulation, gingipain activity was measured in *uvrA* and *pcrA* mutants (Figure 3). In comparison with the wild-type *P. gingivalis* W83 strain, Rgp activity was decreased by approximately 20% in the FLL145 (Δ*uvrA*) mutant strain but there was no significant difference in Kgp activity. Rgp activity was reduced in FLL146 (Δ*pcrA*), FLL147 (Δ*uvrA*-T) and FLL148 (Δ*uvrA*-R) mutants by approximately 60%, 80% and 90% respectively. Kgp activities in FLL146 (Δ*pcrA*), FLL147 (Δ*uvrA*-T) and FLL148 (Δ*uvrA*-R) mutants were greatly reduced by approximately 70%, 90% and 84%. We observed that the FLL147 (Δ*uvrA*-T) and FLL148 (Δ*uvrA*-R) mutants showed the greatest reduction in proteolytic activity.

### Growth in the presence of hydrogen peroxide and sensitivity to UV irradiation

In the inflammatory environment of the mouth, reactive oxygen species (ROS) constitute an important component [13]. An increase in ROS or depletion of antioxidant molecules and/or enzymes results in oxidative stress. ROS can cause damage to cell membranes, nucleic acids, and proteins [48]. While oxidant-induced DNA damage generates over 20 different oxidatively altered bases [49], 8-oxoG is the major product of DNA oxidation [50]. In a previous report we determined that the inactivation of the *uvrB* gene does not affect the sensitivity of *P. gingivalis* to hydrogen peroxide and that the *uvrB*-deficient mutant, *P. gingivalis* FLL144, was significantly more sensitive to UV irradiation than the wild-type [28]. Since there are two *uvrA* paralogs that share 42% homology in *P. gingivalis*, it is likely that one is associated with nucleotide excision repair (NER) mechanism, which is mediated by the products of the following genes: *uvrABC*, *uvrD*, *polA* and *lig* [25], [26] and the other to be a part of the *uvrA-PG1037-pcrA* operon. We therefore evaluated the relationship between the growth of *P. gingivalis* W83 and the isogenic mutants in hydrogen peroxide and documented their sensitivity to UV irradiation to see if *uvrA-PG1037-pcrA* operon was involved in oxidative stress resistance or if recovery from DNA damage was reduced.

**Figure 4:**
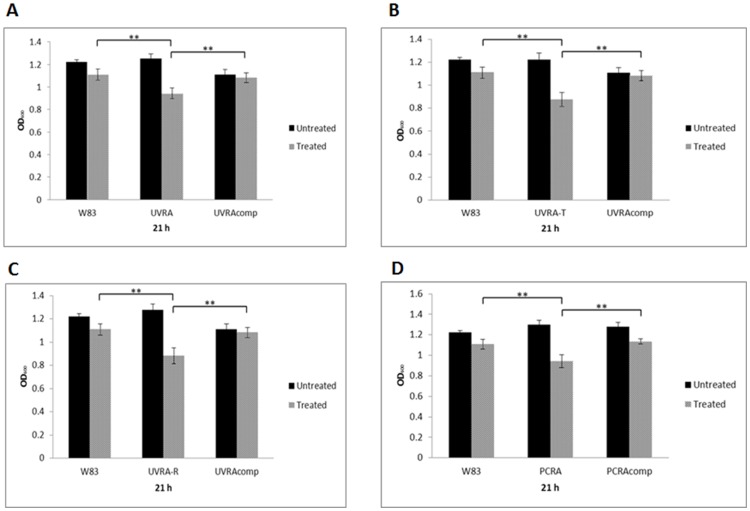
Sensitivity of *P. gingivalis* mutants to hydrogen peroxide. *P. gingivalis* was grown to early log phase (OD_600_ of 0.2) in BHI broth. 0.25 mM H_2_O_2_ was then added to the cell cultures and further incubated over 30 h. Cell cultures without H_2_O_2_ were used as controls. The greatest observable difference in growth rate and response to H_2_O_2_ was seen at 21 h (exponential phase of the growth curve). The results shown are representative of 3 independent experiments each in triplicate. Error bars represent standard error of the mean. **P≤0.01. Asterisks without brackets represent comparisons to treated controls.

**Figure 5:**
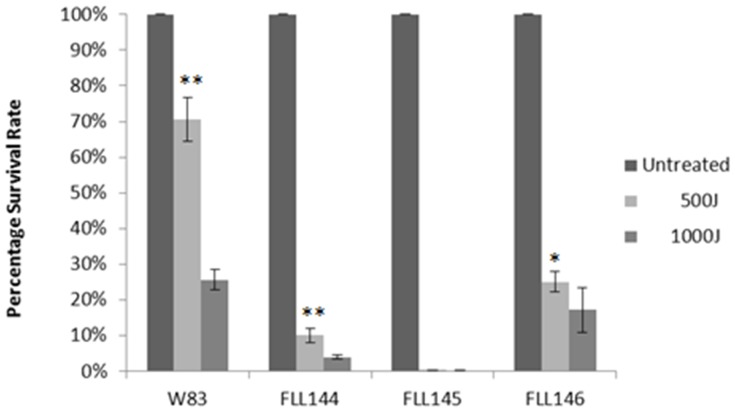
UV sensitivity of *P. gingivalis* mutants. *P. gingivalis* strains W83, FLL144, FLL145 and FLL146 were grown to mid log phase (OD_600_ of 0.6) spread on BHI plates then subjected to irradiation at increasing doses (0 µJ, 500 µJ and 1000 µJ) of UV in a Stratalinker 2400 (Stratagene, La Jolla, CA). **P≤0.01, *P≤0.05.

While the parent strain and *P. gingivalis* FLL144 (*uvrB::ermF-ermAM*) showed similar profiles of sensitivity to 0.25 mM concentrations of hydrogen peroxide [28], *P. gingivalis* FLL145 (Δ*uvrA*), FLL146 (Δ*pcrA*), FLL147 (Δ*uvrA*-T) and FLL148 (Δ*uvrA*-R), showed markedly increased hydrogen peroxide sensitivity (Figure 4). Complementation of the *P. gingivalis uvrA* and *pcrA*-defective mutants restored growth rates to wild type levels. Additionally, *P. gingivalis* FLL145 (Δ*uvrA*) and FLL146 (Δ*pcrA*) showed increased sensitivity to UV irradiation when compared to the wild-type strain (Figure 5). Collectively, these data suggests that the *uvrA-PG1037-pcrA* operon may play a role in H_2_O_2_-induced oxidative stress resistance in *P. gingivalis* and that the inactivation of genes in this operon had dramatic effects on UV sensitivity.

### PG1037 codes for a 67 kDa protein

*PG1037* was cloned into pET102/D-TOPO® expression plasmid (Invitrogen, Carlsbad, CA). The plasmid carrying the gene without mutations was then transformed into competent *E. coli* BL21(DE3)pLysS competent cells and induced for 4 h with IPTG. Lysed *E. coli* cells carrying the rPG1037 was purified then analyzed by SDS-PAGE and stained for the presence of the recombinant protein. Uninduced samples were used for the controls (data not shown). It was determined that *PG1037* codes for the expected 67 kDa recombinant protein, the identity of which was confirmed by Mass Spectrometry and Immunoblot analysis [51].

### Bioinformatic and *In silco* analysis of PG1037

**Figure 6:**
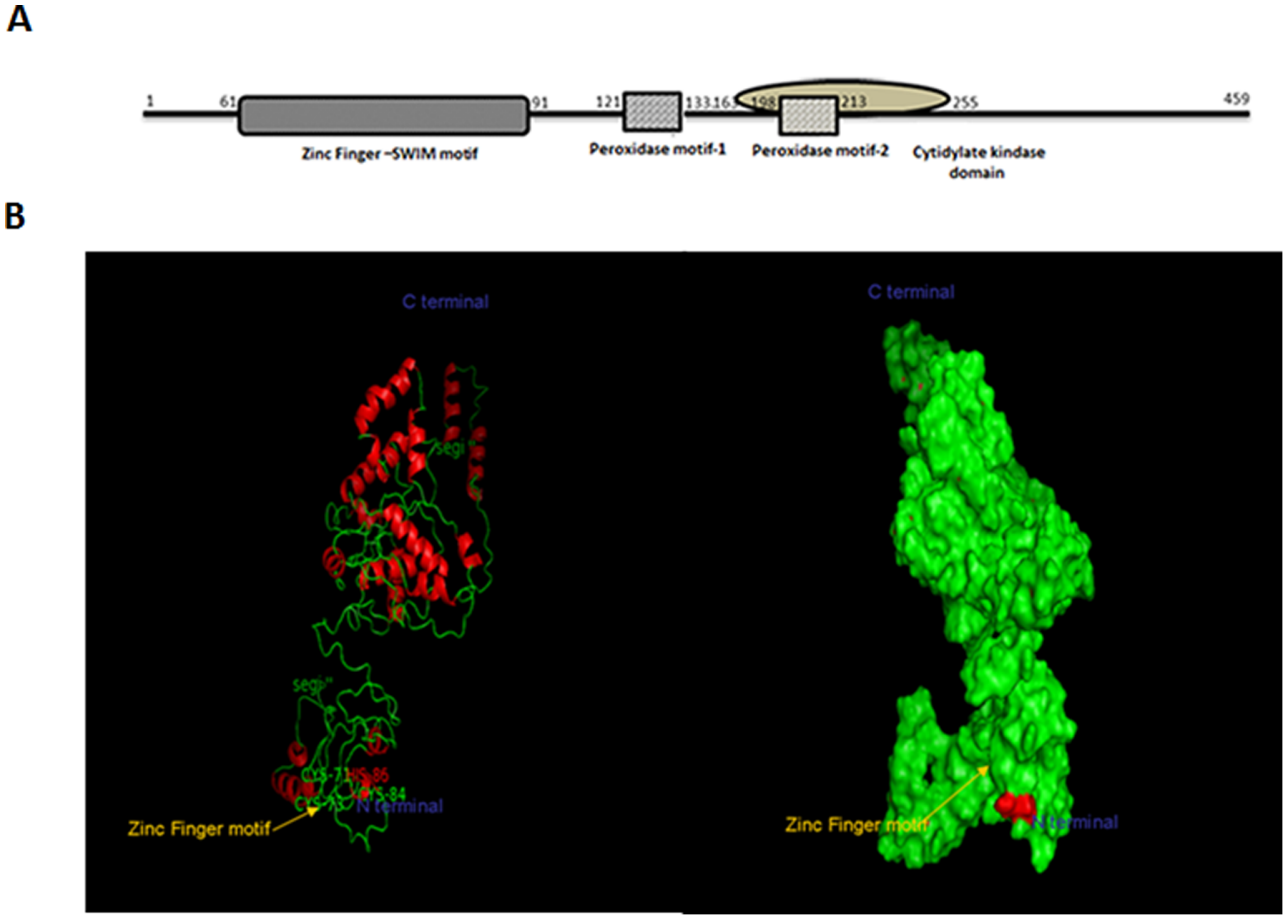
The domain architecture of PG1037. A: Domain architecture of PG1037 shows a ZINC finger – SWIM domain that can interact with DNA, proteins and also may also be involved in reducing oxidative lesions in the DNA. This is followed by two peroxidase motifs which scavenges the peroxide radicals. The cytidylate kinase domain flanking the second peroxidase motif, could be involved in the transferring of phosphate groups. B: The Zinc finger attachment site in PG1037.

Bioinformatic analysis revealed that PG1037 is a cytoplasmic protein. *In silico* analysis of the conserved hypothetical protein revealed that it contained three conservative domains, one representing the zinc finger domain, two peroxidase homologous motifs and a cytidylate kinase domain (Figure 6.A.). The amino acid sequence of PG1037 showed conserved repeats which were found in the helical structures of the protein and could be assumed to have a significant role in protein-protein interaction. PG1037 contains the characteristic Zinc Finger – SWIM motif which was predicted to be organized in the N-terminal beta strands possibly adopting the 4 Cysteine structure anchoring the Zinc ion (Figure 6.B). The Zinc ion could play a role as a metal catalyst for the enzymatic action of the protein in forming a reduced compound terminally combating oxidative stress.

### 8-oxoG repair activity is similar in both wild-type and mutant *P. gingivalis* strains

**Figure 7:**
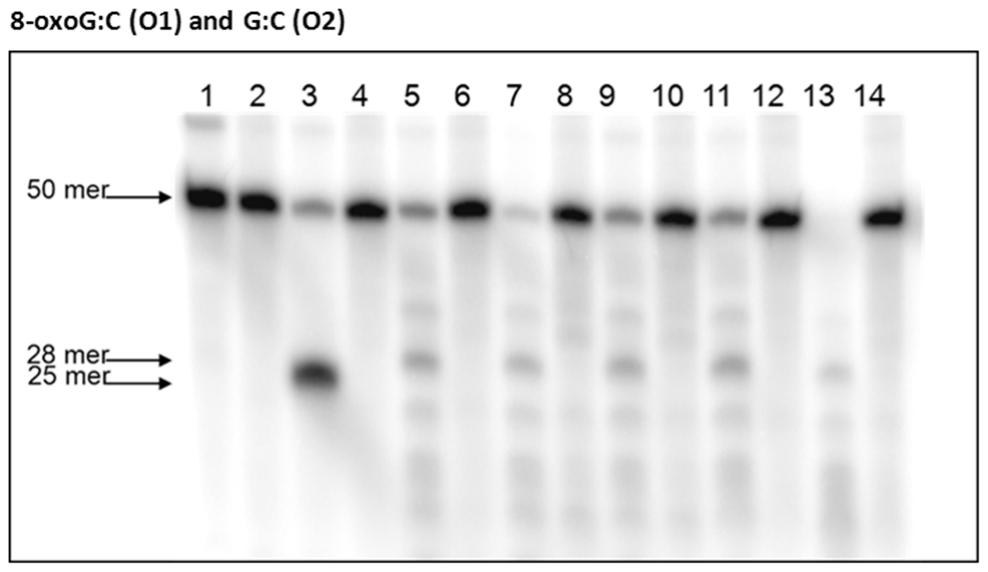
8-oxoG removal activities of cell extracts from *P. gingivalis* strains W83 and isogenic mutants. [γ-^32^P]-ATP-5′-end-labeled oligonucleotides (O1 and O2) were incubated with *P. gingivalis* extracts for 10 min, electrophoresed and visualized. Lane 1 contained O1; Lane 2 contained O2; Lane 3 contained O1 and Fpg enzyme; Lane 4 contained O2 and Fpg enzyme; Lane 5 contained O1 and *P. gingivalis* W83 extract; Lane 6 contained O2 and *P. gingivalis* W83 extract; Lane 7 contained O1 and *P. gingivalis* FLL145 extract; Lane 8 contained O2 and *P. gingivalis* FLL145 extract; Lane 9 contained O1 and *P. gingivalis* FLL146 extract; Lane 10 contained O2 and *P. gingivalis* FLL146 extract; Lane 11 contained O1 and *P. gingivalis* FLL147 extract; Lane 12 contained O2 and *P. gingivalis* FLL147 extract; Lane 13 contained O1 and *P. gingivalis* FLL148 extract and Lane 14 contained O2 and *P. gingivalis* FLL148 extract.

Previously in our lab, we had established that the removal of 8-oxoG was not by a nucleotide excision repair mechanism (NER) but by some other yet-to-be defined mechanism [28]. Since the *uvrA-PG1037-pcrA* operon seemed to have some involvement in oxidative stress resistance and UV sensitivity, *P. gingivalis* W83 and the isogenic mutants defective in PG1036 and PG1038 were assessed for enzymatic removal of 8-oxoG. Bacterial extracts from *P. gingivalis* W83 and isogenic strains grown in the presence or absence of hydrogen peroxide, were used in glycosylase assays with a [γ-^32^P]-ATP-5′-end-labeled 8-oxodG:C-containing oligonucleotide (50-mer) [Table 3]. As shown in Figure 7, *E. coli* Fpg enzyme generated a 25 mer cleavage fragment while a 28 mer cleavage product was observed in *P. gingivalis* strains W83 and isogenic mutants. Similar results were observed when extracts were treated with or without hydrogen peroxide. As a control (not shown), the removal of uracil was examined using the same extracts. The level of activity for Ung was similar in all *P. gingivalis* strains. This data suggest that the *uvrA* and the *-pcrA* genes might not be directly involved in the removal of 8-oxoG.

### 8-oxoG repair activity is reduced in the presence of rPG1037

**Figure 8:**
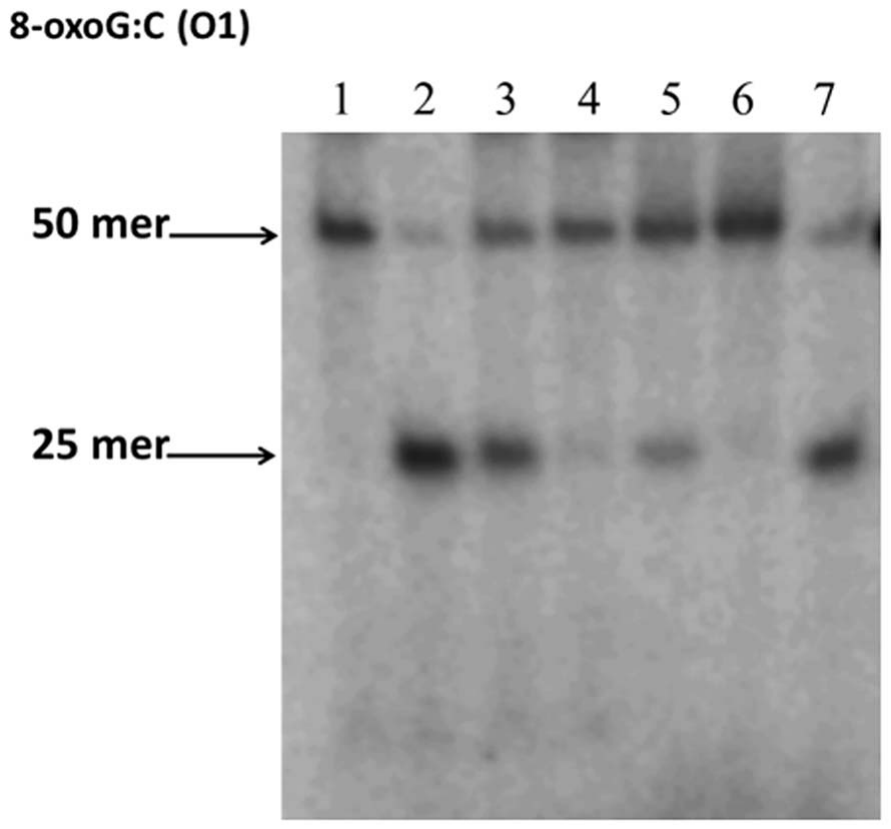
8-oxoG removal activities of Fpg enzyme. [γ-^32^P]-ATP-5′-end-labeled oligonucleotides (O1) was incubated with rPG1037 for 20 min, H_2_0_2_ for 10 min then Fpg enzyme for 30 min, electrophoresed and visualized. Lane 1 contains O1; Lane 2 contains O1 and Fpg enzyme; Lane 3 contains O1, 1 µg rPG1037 and Fpg enzyme; Lane 4 contains O1, 2 µg rPG1037 and Fpg enzyme; Lane 5 contains O1, 1 µg rPG1037, 2 mM H_2_0_2_ and Fpg enzyme; Lane 6 contains O1, 2 µg rPG1037, 2 mM H_2_0_2_ and Fpg enzyme; Lane 7 contains O1, Fpg enzyme and 2 mM H_2_0_2_.

Because the rPG1037 protein can bind the 8-oxoG lesion [28], we investigated whether this protein would have an effect on its enzymatic removal or repair. [γ-^32^P]-ATP-5′-end-labeled 8-oxodG:C-containing oligonucleotide (50-mer) [Table 3] was incubated with rPG1037 in the presence or absence of hydrogen peroxide. The addition of the *E. coli* Fpg enzyme resulted in reduced cleavage of the lesion in the presence of rPG1037 under oxidative conditions (Figure 8). In the presence of rPG1777 (14 kDa) and rPG0686 (62 kDa), two other *P. gingivalis* proteins not known to bind the 8-oxoG lesion, the cleavage of the lesion by Fpg was unaffected in the presence or absence of hydrogen peroxide (data not shown).

### rPG1037 protects DNA from Fenton Chemistry-mediated DNA damage

**Figure 9:**
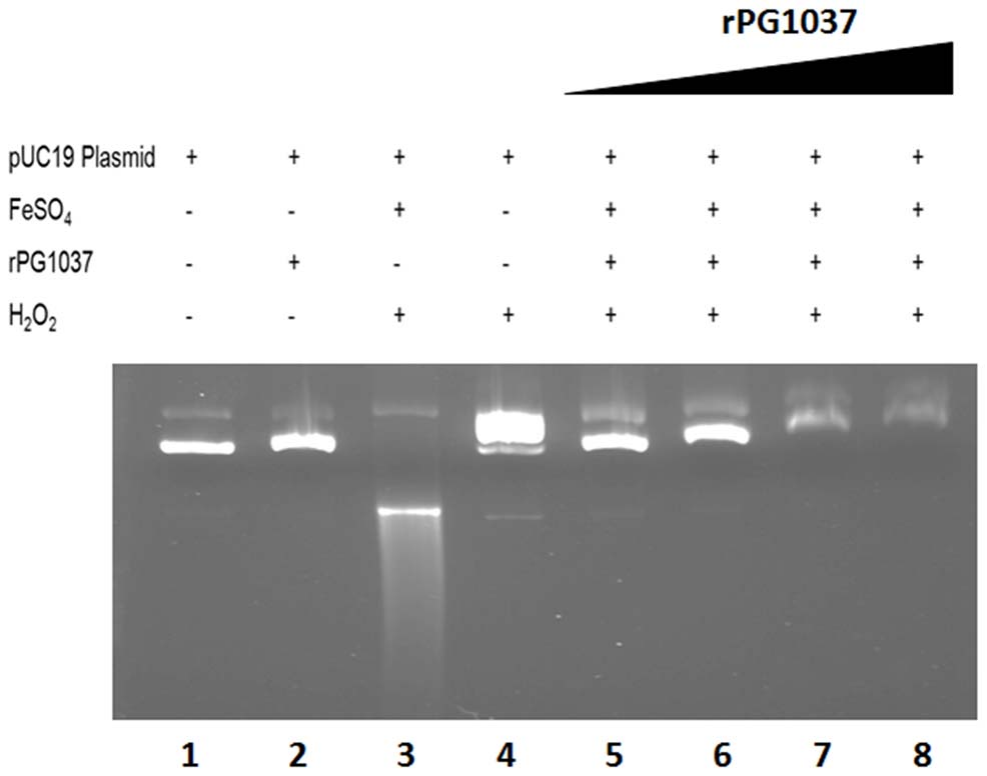
rPG1037 prevents Fenton chemistry-mediated DNA damage in *vitro*. The 1% agarose gel shown was loaded with various reaction components, with protein concentrations increasing as indicated. Lane 1 contains pUC19 plasmid; Lane 2 contains pUC19 and rPG1037 only; Lane 3 shows degradation of the pUC19 plasmid in the presence of FeSO_4_ and H_2_O_2_; Lane 4 shows degradation of the pUC19 plasmid in the presence of H_2_O_2_ only; Lanes 4–8 shows that increasing concentrations of rPG1037 (1 µg, 2 µg, 3 µg and 4 µg) abrogates the effect of FeSO_4_ and H_2_O_2_ and results in retardation of pUC19 plasmid through the agarose gel. When present, FeSO_4_ was at 50 µM and hydrogen peroxide was at 880 mM final concentration.

Free iron in cellular systems can interact with free radicals such as hydrogen peroxide to generate hydroxyl radicals that can attack and damage DNA [52]. We assessed whether PG1037 may play a role in binding and preventing damage to cellular DNA. DNA damage by hydroxyl radicals was assessed *in vitro* by monitoring the degradation of supercoiled pUC19 plasmid DNA in the presence of Fe(II), hydrogen peroxide and increasing concentrations of the rPG1037 protein. In the presence of rPG1037, the pUC19 DNA was protected from degradation when exposed to hydrogen peroxide in the presence of iron (Figure 9).

### rPG1037 forms a complex with other proteins

We have previously shown that a protein complex interacts with 8-oxoG lesions [28]. We identified the PG1037 protein as a major interacting partner, but to date the mechanism of interaction and what role it plays in the removal of 8-oxoG is still unclear. To further elucidate the function of PG1037, the purified rPG1037 protein was attached to Ni-NTA-linked magnetic beads and incubated with cell lysates from *P. gingivalis* W83 or FLL92 (*vimA::ermF-ermAM*) exposed to oxidative stress. As a negative control, the lysates were incubated with the magnetic beads without the recombinant protein. The extracted protein was separated by SDS-PAGE and identified by mass spectrometry. As summarized in Table 4, PG1037 can interact with several other proteins that have DNA binding or oxidoreductase activities.

**Table 4:** Proteins that interact with recombinant PG1037.

Gene ID	Gene name	Molecular Weight	Functional class	Molecular Function
PG1803	V-type ATP synthase subunit A	65 kDa	Energy metabolism; ATP-proton motive force	ATP binding, hydrogen ion transporting ATP synthase activity
PG1401	Tryptophanse	52 kDa	Energy metabolism; Amino acids to amines	Catalytic Activity; Lyase Activity; Carbon-Carbon lyase activity; Pyridoxal phosphate binding
PG1385*	Conserved hypothetical protein	46 kDa	Unknown; Conserved hypothetical	Binding
PG1323	phoH-related protein	50 kDa	Uncategorized	ATP binding
PG1279	D-3-phosphoglycerate dehydrogenase	33 kDa	Amino acid biosynthesis; Serine family	Oxidoreductase activity; Cofactor binding, NAD binding
PG1082	Phosphotransacetylase	35 kDa	Energy metabolism	Acyltransferase activity; Acetyltransferase activity
PG1080*	3-hydroxybutyryl-CoA dehydrogenase	30 kDa	Fatty acid and phospholipid metabolism	Oxidoreductase activity; Coenzyme binding
PG1076	Butyryl-CoA dehydrogenase	42 kDa	Fatty acid and phospholipid metabolism	Acyl-CoA dehydrogenase activity; Oxidoreductase activity, acting on the CH-CH group of donors; FAD binding
PG1069	Zinc-containing alcohol dehydrogenase	37 kDa	Energy production and conversion	NADPH:quinone reductase and related Zn-dependent oxidoreductase
PG0689**	Iron-containing alcohol dehydrogenase	42 kDa	Energy Metabolism	Oxidoreductase activity; Metal ion binding
PG0593	Periplasmic serine protease	53 kDa	Protein fate; Degradation of proteins, peptides and glygopeptides	Serine-type endopeptidase activity; Protein binding
PG0548	Pyruvate/flavodoxin oxidoreductase	60 kDa	Energy metabolism; Electron transport	Oxidoreductase activity; iron-sulfur cluster binding; Iron ion binding
PG0514	Preprotein translocase subunit A	126 kDa	Protein Fate; Protein and peptide secretion and trafficking	Nucleic acid binding; Helicase activity; Protein binding; ATP binding
PG0414	Conserved hypothetical protein	72 kDa	Unknown; Conserved hypothetical	
PG0394	DNA-directed RNA polymerase subunit beta	142 kDa	Transcription; DNA dependent RNA polymerase	DNA binding; DNA-directed RNA polymerase activity
PG0121	DNA-binding protein (HU-related)	9 kDa	DNA metabolism; Chromosome-associated proteins	DNA binding

* Proteins from W83 that interacted with PG1037 only.

** Proteins from FLL92 and W83 that interacted with PG1037.

## Discussion

DNA damage is a major consequence of oxidative stress. Several conserved mechanisms observed in other organisms that can act cooperatively in the removal of damaged nucleotides or repair DNA mispairing resulting from oxidative stress, are also present in *P. gingivalis* [53]–[56]. However, the repair of oxidative stress-induced DNA damage involving 8-oxoG may occur by a still undescribed mechanism in *P. gingivalis*. Elucidation of this mechanism could reveal how *P. gingivalis* survives the challenge of oxidative stress typical of the inflammatory microenvironment in the periodontal pocket during periodontitis. In this study, we evaluated the role of the *uvrA-PG1037-pcrA* operon in the repair of oxidative stress-induced DNA damage in *P. gingivalis*.

Protection against oxidative damage utilizes a unique mechanism in *P. gingivalis*. Cell surface heme acquisition has been shown to be a defense mechanism against ROS in *P. gingivalis* [57], [58]. The storage of the heme on the cell surface which gives the organism its characteristic black pigmentation, can form μ-oxo dimers in the presence of ROS and can give rise to the catalytic degradation of H_2_O_2_ [57]. The gingipains are known to play a significant role in hemin acquisition [59], [60], binding, and accumulation in *P. gingivalis* [60]–[63]. Reduced gingipain activity and a non-pigmented phenotype in *P. gingivalis* can result in elevated 8-oxoG levels in the genome and increased sensitivity to H_2_O_2_-induced oxidative stress [23]. The *uvrA* and *pcrA*-defective isogenic mutants had reduced gingipain activities but its relative significance in the sensitivity to H_2_O_2_-induced oxidative stress is unclear because these mutants all had a black-pigmented phenotype. Because the mechanism of the modulation of gingipain activity is unknown in these strains, we cannot rule out a common regulatory pathway that may directly or indirectly affect the level of sensitivity to H_2_O_2_-induced oxidative stress.

In studies using oligonucleotides bound to Streptavidin magnetic beads, several proteins were identified that specifically interacted with the 8-oxoG lesion [28]. One of these proteins (PG1037) was encoded for by a gene that is part of the *uvrA-PG1037-pcrA* transcriptional unit (Figure 1). In *P. gingivalis*, this transcriptional unit confirmed in this study, was upregulated under H_2_O_2_-induced oxidative stress [28], [64]. Functional homologues of UvrA and PcrA a putative helicase, are known to be involved in DNA repair [64]–[66]. In NER, UvrA, UvrB and UvrC proteins recognize and cleave the damaged DNA in a multistep reaction [67], [68]. The UvrAB heterodimer scans the DNA searching for large distortions in the helix such as ones caused by pyrimidine dimers [69]. Once a damaged site is found, UvrA dissociates, and a stable UvrB-DNA complex is formed. UvrC associates to bind UvrB and enables the UvrB protein to nick the DNA at the fourth nucleotide 3′ to the site of damage. Following the 3′ incision, UvrC protein catalyzes nicking of the DNA at the seventh nucleotide, 5′ to the damage. The damaged fragment is removed by a helicase (UvrD). The remaining gap is filled in by the DNA polymerase and the nick sealed by a DNA ligase.

In *P. gingivalis*, there are two *uvrA* paralogues that share 42% homology (http://www.ncbi.nlm.nih.gov/). One of these paralogues is associated with the NER system [25], [26] while the other is a part of the *uvrA-PG1037-pcrA* operon. Similar to other organisms such as *Heamophilus influenza* [70], *Streptococcus mutans* [71], *Bacillus subtilis* [65] and *Plasmodium falciparum* [72], our *P. gingivalis uvrA* (FLL145) and *P. gingivalis pcrA* (FLL146) defective isogenic mutants showed extreme sensitivity to UV irradiation. The functions of these genes appear to be conserved as defects in their homologues in other organisms showed a similar phenotype [65], [70]–[72]. Additionally, when an essential component of the NER system in *P. gingivalis* was interrupted, creating the *uvrB*-defective mutant (FLL144), this isogenic strain was more sensitive to UV irradiation than the wild-type [28]. The *P. gingivalis uvrA*-defective mutant (FLL145) and *P. gingivalis pcrA*-defective (FLL146) mutant, also showed sensitivity to oxidative stress compared to the wild-type which suggests that these genes are not only involved in repair to DNA damage caused by UV irradiation but are also integral in oxidative stress resistance. This is undoubtedly unique as the *uvrB*-defective mutant (FLL144) in which both *uvrA* paralogues were intact, and the *uvrA-* and *pcrA-* defective mutants in other organisms showed no obvious involvement in sensitivity to oxidative stress.

Repair of oxidative stress-induced DNA damage was similar in the wild-type *P. gingivalis* W83 and isogenic mutants defective in the *uvrA* and *pcrA* genes. While these gene products can play a role in oxidative stress resistance in *P. gingivalis* it is likely that they may not be involved in the repair of the 8-oxoG lesion. Another possibility is that the presence of other functional homologues (e.g. the UvrA paralogue) may complement these defects. Furthermore we cannot rule out the presence of PG1037 in the *uvrA-* and *pcrA-*defective isogenic mutants. The inability to inactivate this gene with unknown function suggests that it is essential. This is consistent with a recent report using a Mariner transposon system that identified *PG1037* as one of the essential genes in *P. gingivalis* [73]. It is likely that *PG1037* is deferentially expressed and that PG1037 could have multiple functions, including the protection of oxidative stress-induced DNA damage, since this conserved hypothetical protein contains a zinc finger domain, two peroxidase homologous motifs and a cytidylate kinase domain.

Zinc fingers are a common structural element utilized by sequence-specific DNA-binding proteins to interact with DNA [74]. Additionally, zinc fingers can also mediate protein-protein, protein-RNA, and protein-ligand interactions [75]. Since PG1037 possesses a zinc finger motif near the N terminus on its beta strand and shows a signature of Cys-X-Cys-X_2_-Phe-X_4_-Leu-X_2_-Cys-X-His spanning positions 61 to 91, this gives PG1037 a versatile domain that can interact with DNA or proteins in different contexts [Figure 6.A.] [76]. It is possible that the presence of this domain gives PG1037 the unique capability to interact with several protein partners demonstrated by its interaction with the 8-oxoG lesion [28] and various other proteins with DNA binding and oxidoreductase properties (Table 4). Additionally, PG1037 contains two peroxidase motifs which may be involved in the detoxification of hydrogen peroxide or other reactive oxygen species. Our data showed that not only did the recombinant PG1037 have the ability to prevent Fenton chemistry-mediated DNA damage in *vitro* but its presence reduced the cleavage of the 8-oxoG lesion by Fpg under oxidative stress conditions. Because we cannot completely rule out steric hindrance of Fpg by rPG1037, the direct involvement of PG1037 in the repair of 8-oxoG is still unclear. Its putative structure however, raises questions about its ability to protect DNA from oxidative stress-induced DNA damage. In a likely scenario, it is possible that PG1037, which contains positively charged arginine at positions 62, 65, 68 and 77 upstream from the zinc finger motif on the beta strand, could interact with the phosphate group of the lesion. A conformational change in the protein due to oxidative stress could bring about the cysteine and histidine in the zinc finger motif to form a unique trap that could interact with the double bond at oxygen of the 8-oxoG lesion. This reduction reaction could cleave the double bond, setting free the singlet oxygen to be either reduced by the zinc finger itself by the reducing activity of cysteine, or be reduced by the action of peroxidase motifs in the protein. A cytidylate kinase domain found further in the protein could also act in transferring the phosphate group after the breakdown of the lesion [77]. Based on the domain architecture of PG1037 and its primary function as a zinc finger protein, we can postulate that its involvement in repair of oxidatively damaged bases is highly likely and it is possible that the presence of peroxidase motifs and a cytidylate domain would aid in the detoxification and resolution of damage caused by harmful peroxide radicals. This hypothesis is currently under further investigation in the laboratory.

The *uvrA-PG1037-pcrA* operon is undoubtedly unique and the data from this study supports our hypothesis that complex mechanisms are involved in oxidative stress resistance and the removal of 8-oxoG in *P. gingivalis*. Further investigation is needed to delineate the role of this operon in response to an oxidative insult in *P. gingivalis.*
